# The Cancer Microbiota: EMT and Inflammation as Shared Molecular Mechanisms Associated with Plasticity and Progression

**DOI:** 10.1155/2019/1253727

**Published:** 2019-10-20

**Authors:** Daniele Vergara, Pasquale Simeone, Marina Damato, Michele Maffia, Paola Lanuti, Marco Trerotola

**Affiliations:** ^1^Department of Biological and Environmental Sciences and Technologies, University of Salento, Lecce, Italy; ^2^Laboratory of Clinical Proteomic, “Giovanni Paolo II” Hospital, ASL-Lecce, Italy; ^3^Department of Medicine and Aging Sciences, “G.d'Annunzio” University of Chieti-Pescara, Chieti, Italy; ^4^Laboratory of Cytomorphology, Center for Advanced Studies and Technology (CAST), “G.d'Annunzio” University of Chieti-Pescara, Chieti, Italy; ^5^Laboratory of Cancer Pathology, Center for Advanced Studies and Technology (CAST), “G.d'Annunzio” University of Chieti-Pescara, Chieti, Italy; ^6^Department of Medical, Oral and Biotechnological Sciences, “G.d'Annunzio” University of Chieti-Pescara, Chieti, Italy

## Abstract

With the advent of novel molecular platforms for high-throughput/next-generation sequencing, the communities of commensal and pathogenic microorganisms that inhabit the human body have been defined in depth. In the last decade, the role of microbiota-host interactions in driving human cancer plasticity and malignant progression has been well documented. Germ-free preclinical models provided an invaluable tool to demonstrate that the human microbiota can confer susceptibility to various types of cancer and can also modulate the host response to therapeutic treatments. Of interest, besides the detrimental effects of dysbiosis on cancer etiopathogenesis, specific microorganisms have been shown to exert protective activities against cancer growth. This has strong clinical implications, as restoration of the physiologic microbiota is being rapidly implemented as a novel anticancer therapeutic strategy. Here, we reviewed past and recent literature depicting the role of microbiota-host interactions in modulating key molecular mechanisms that drive human cancer plasticity and lead to malignant progression. We analyzed microbiota-host interactions occurring in the gut as well as in other anatomic sites, such as oral and nasal cavities, lungs, breast, esophagus, stomach, reproductive tract, and skin. We revealed a common ground of biological alterations and pathways modulated by a dysbiotic microbiota and potentially involved in the control of cancer progression. The molecular mechanisms most frequently affected by the pathogenic microorganisms to induce malignant progression involve epithelial-mesenchymal transition- (EMT-) dependent barrier alterations and tumor-promoting inflammation. This evidence may pave the way to better stratify high-risk cancer patients based on unique microenvironmental/microbial signatures and to develop novel, personalized, biological therapies.

## 1. Introduction

The human microbiota is defined as the population of microorganisms residing on or within the human body sites. The collective genome content of the human microbiota (the microbial metagenome) is known as the human microbiome, although the words “microbiome” and “microbiota” are often used interchangeably [1]. The total number of cells in the “reference” human being (30-year-old young adult, weighing 70 kg, 1.72 m tall, and with a body area of 1.85 m^2^) has been estimated to be about 3.7 × 10^13^ [2, 3]. The estimate of the number of microbial cells in the “reference” human body was recently revised and reported to be approximately 3.9 × 10^13^, with a bacterial (B)-to-human (H) ratio close to 1 [4]. Despite the abundance of microbial cells in the human body, they have been estimated to account only for the 2-7% of the individual's biomass (excluding water weight) given their small dimensions [5]. However, the microbiome encodes about 100-fold more genes than the human genome, suggesting a strong impact on the physiology of the human body [5]. The metabolic activity of the microbiota can exert profound effects on the organism, mostly beneficial in the eubiosis state; in case of dysbiosis, though, the altered microbiota can have detrimental effects and may be strongly related to the pathogenesis of several human diseases [6–8].

**Figure 1:**
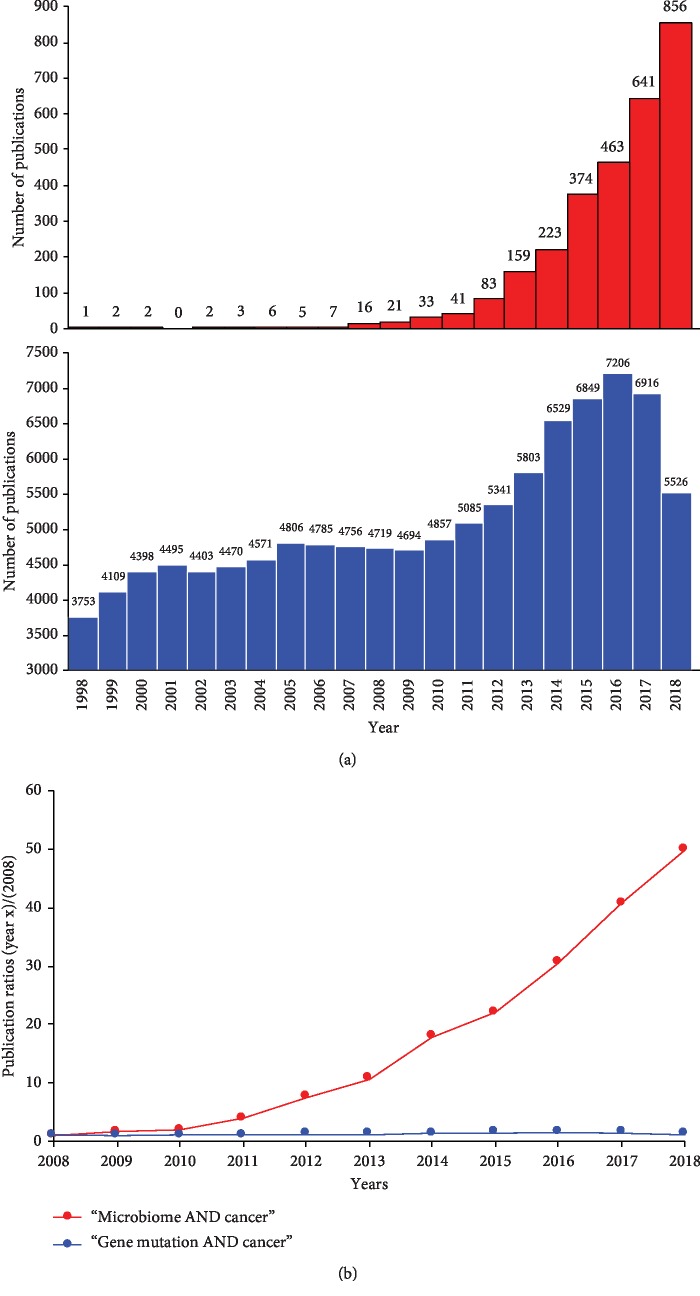
The importance of the microbiota in cancer. (a) Bar graphs showing the number of manuscripts published between 1998 and 2018, as retrieved by interrogation of the PubMed bibliographic database using the keywords “microbiome AND cancer” (red, top) and “gene mutation AND cancer” (blue, bottom). (b) Graph showing the growth rate of the “microbiome AND cancer” topic (red) *vs* the “gene mutation AND cancer” topic calculated in the last 10 years. Each point on the curve represents the ratio between the number of publications in the indicated year and the number of publications in 2008.

Major milestones have been reached in the last decade by the Human Microbiome Project (HMP). This is a two-phase research initiative aimed at identifying and characterizing the whole human microbiota through the extensive use of metagenomics and whole genome sequencing (first phase, HMP1, launched in 2007), and at elucidating the role of microbes in human diseases through the use of multiple “omics” technologies (second phase, Integrative HMP, launched in 2014) [9, 10]. In 2012, the microbiome of healthy humans was mapped and a reference database was created [9]. Mutations in key driver genes — such as those that inactivate genes responsible for DNA repair — are a primary cause of cancer pathogenesis, although the HMP supported the idea that a dysbiotic microbiota can substantially contribute to cancer progression. We carried out a bibliographical search in the PubMed database using the keywords “microbiome AND cancer” and “gene mutation AND cancer” (Figure 1(a)). Comparison of the number of publications in the last 10 years revealed a strikingly higher growth rate of the topic “microbiome AND cancer” *vs* “gene mutation AND cancer” (Figure 1(b)). Thus, the microbiota is quickly becoming a rising star due to its role in the modulation of malignant progression. Moreover, the affordable costs of high-throughput technologies such as genomics, transcriptomics, proteomics, metabolomics, and epigenomics have boosted the interests of the scientific community towards a deep characterization of the host-microbiota interactions and of the mechanisms underlying their dysfunctions in human diseases, in order to develop new therapeutic strategies. On the other hand, it remains highly debated whether dysbiosis can play a causative role in carcinogenesis or rather it is an effect of tumor development. Unlike viruses, which express constitutively active viral mimics of cellular proto-oncogenes, tumorigenesis associated with microbial dysbiosis can arise after multiple hits. The recent technological advancements in the use of gnotobiotic (including germ-free) mouse models helped to demonstrate that the microbiota can alter cancer susceptibility and progression by modulating metabolism and inflammation [5], whose alterations are recognized hallmarks of cancer [11]. In this context, the most relevant evidence about a causative role of the microbiota in tumorigenesis comes from several studies demonstrating that *Helicobacter pylori* (*H. pylori*) is the etiologic agent of gastritis and gastric ulcers, which can be precursors of gastric adenocarcinoma [12].

Here, we reviewed the most important evidence obtained in the understanding of the microbiota-host interaction mechanisms that drive human cancer plasticity and lead to malignant progression. Most studies aimed at elucidating the effects of microbiota-host interactions on human cancer pathogenesis have been focused on colorectal cancer (CRC), for the obvious reason that the greatest number and diversity of microbes in the human body inhabits the large intestine (10^12^ bacteria/gm stool) [13]. However, many experimental findings have been collected on the links between dysbiosis and development of cancer in anatomic sites outside the gut, including oral and nasal cavities, lungs, skin, breast, and reproductive tract. Many of the same pathways that mediate the interplay between host microbiota, inflammation, and cancer etiology in the intestine may be applicable to other malignancies, particularly those that develop in organs directly communicating with the gastrointestinal tract. For example, bacteria can transform ingested material into toxic metabolites or secrete toxic substances, contributing to promote inflammation-dependent carcinogenesis outside their primary sites of colonization. The discussion points of this review will be developed as subsections corresponding to the anatomic sites where specific microbial communities reside. A common ground of signaling mechanisms responsible for the induction of plasticity either in cancer cells or in the surrounding niches is finally proposed.

## 2. Oral Cavity

Oral cancer, primarily oral squamous cell carcinoma (OSCC) arising from the oral mucosa, is caused by both genetic and environmental factors, such as tobacco and alcohol consumption, betel quid chewing, and human papillomavirus infections [14]. However, approximately 15% of oral cancer cases cannot be attributed to these major risk factors and are potentially induced by altered oral bacterial communities [14]. The oral cavity of healthy individuals is inhabited by a multispecies microbiota that usually exists in a balanced immunoinflammatory state with the host [15]. Certain species, such as *Porphyromonas gingivalis* (*P. gingivalis*), can induce dysbiosis [16]. In this condition, other microbes, such as *Fusobacterium nucleatum* (*F. nucleatum*), can become opportunistically pathogenic and lead to dysregulated immune response and increased risk to develop periodontal diseases and OSCC [16, 17]. Other specific bacteria, such as *Streptococcus* sp., *Peptostreptococcus* sp., and *Prevotella* sp., have been identified to correlate strongly with OSCC [18]. *P. gingivalis* is able to stimulate the expression of the cancer stem cell markers CD133 and CD44 [19]. It has been recently demonstrated that prolonged infections of oral cancer cells by *P. gingivalis* promote migratory and invasive properties [19, 20]. The underlying molecular mechanisms have been elucidated and involve increased expression of matrix metalloproteinase- (MMP-) 1 and MMP-10 and induction of the epithelial-mesenchymal transition (EMT) factors Slug, Snail, and Zeb1 without requiring repression of miR-200b or E-cadherin [19, 20]. Moreover, *P. gingivalis* mutant strains lacking the fimbrial protein FimA were attenuated in their ability to activate Zeb1 expression, demonstrating a role for the FimA adhesin in triggering EMT [20].

## 3. Nasal Cavity

The olfactory epithelium microbial community is mainly dominated by 4 phyla: Bacteroidetes, Firmicutes, Proteobacteria, and Actinobacteria [21]. Significant differences in bacterial composition were observed in relation to localized factors, including temperature, humidity, and position in the respiratory tract [22]. Microbiota can modulate expression and functions of critical mediators of the olfactory signal transduction pathways [21]. This profoundly impacts both physiology and function of the olfactory epithelium [23]. In addition to mediating these physiological functions, changes in the microbiota composition are often associated with several immunological diseases of the nasal cavity including allergic rhinitis and chronic rhinosinusitis [22]. Bacterial components may regulate the epithelial barrier functions and promote tissue-remodeling processes [24]. Epithelial cells of the human airways are covered by a thick mucus layer, which is renewed continuously. This mucus layer is fundamental for the mucociliary clearance, which allows inhaled microorganisms and other particles to be removed from the airways [25]. In physiological conditions, it is unlikely that inhaled bacteria reach the apical surfaces of epithelial cells. Viral infections or pathological conditions, such as cystic fibrosis, can lead to dysregulation of the mucociliary clearance [26]; in such cases, microbial commensals of the nasal cavity may prolong their permanence on the mucus surface, forming colonies and secreting soluble virulence-associated factors. It has been recently found that the exposure of human airway epithelial cells to *α*-toxin/hemolysin A from *Staphylococcus aureus* (*S. aureus*) induces improper cytoskeletal remodeling due to destabilization of cell-cell contacts and focal adhesions [27]. This results in the formation of paracellular gaps and enhanced permeability of the epithelial layer [27]. *S. aureus* plays a key role in the pathogenesis of nasal polyposis, through secreted products, such as the protein A that induces mast cell degranulation and the staphylococcal enterotoxins (SAE) that induce release of proinflammatory cytokines [28]. Nasal polyps have been found to express lower levels of the junctional proteins E-cadherin, zonula occludens-1 (ZO-1), and occludin and higher levels of TGF*β* and vimentin as compared with the healthy nasal mucosa [29]. Thus, the loss of epithelial features observed in nasal polyps appears resulting from the activation of an EMT program, and *S. aureus* could play an important role as driver of this epithelial plasticity.

## 4. Lungs

The normal lung was considered as a microbe-free organ for a long time. Over the past decade, novel culture-independent techniques for microbial identification have challenged this dogma and revealed that the lung is constantly exposed to a variety of air-borne microbes through inhalation [30]. The most abundant phyla are Bacteroidetes and Firmicutes, and prominent genera include *Prevotella*, *Veillonella*, and *Streptococcus*. There is large overlap in the community composition between the lung microbiota and that of the oral cavity as compared with other body sites [31]. The number of bacteria in the lungs can markedly increase in the presence of respiratory diseases, due to changes of pH, oxygen tension, temperature, and immune conditions and also to inflammatory events, that are often linked to an increased production of mucus, which in turn represents an important source of microbial nutrients [30]. The molecular mechanisms mediating the effect of a dysbiotic microbiota on lung cancer development include increased genotoxic and virulence effects and altered metabolism, immune response, and inflammation [32]. It has been recently demonstrated that lung adenocarcinoma (LUAD) can activate tissue-resident lymphocytes to establish a protumorigenic microenvironment and that this depends on the local microbiota [33]. As an example, a LUAD mouse model carrying *Kras* mutations and *p53* deletion was utilized to compare germ-free and specific pathogen-free conditions, revealing that germ-free mice are significantly protected against LUAD [33]. The lung microbiota was found to induce myeloid cells to produce interleukin-1*β* (IL-1*β*) and IL-23, and *γδ* T cells to promote inflammation and tumor cell proliferation through IL-17 [33].

## 5. Breast

**Figure 2:**
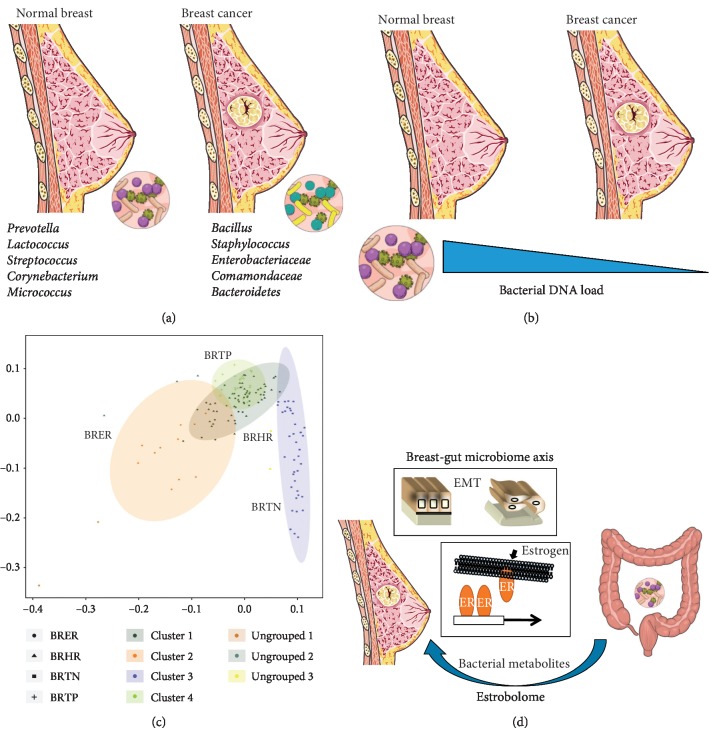
Breast cancer microbiota and the gut-breast crosstalk. (a) Different bacterial profile between healthy women and breast cancer patients. (b) Bacterial DNA load is reduced in tumor versus paired normal and healthy breast tissues. (c) Hierarchical clustering of endocrine receptor (estrogen or progesterone receptor) positive (BRER), human EGFR2 (HER2) positive (BRHR), triple positive (estrogen, progesterone, and HER2 receptor positive) (BRTP), and triple negative (absence of estrogen, progesterone, and HER2 receptors) (BRTN) tumors based on their microbial signature. Modified from [35]. (d) Factors and mechanisms by which gut microbes influence breast cancer development and progression. Gut microbes can affect breast physiology by producing specific metabolites that can activate the EMT program or influence the metabolism of estrogens with a potential impact on estrogen receptor-positive tumor subtypes. Panels (a), (b), and (d) have been modified from Servier Medical Art, licensed under a Creative Common Attribution 3.0 Generic License; http://smart.servier.com/.

Studies performed on human samples have demonstrated that different bacterial profiles can be detected in the normal adjacent breast tissue from breast cancer patients and in normal tissues from healthy controls [34] (Figure 2(a)). This is also associated with a significantly reduced amount of bacterial DNA load in breast tumors *vs* paired normal adjacent tissues [34] (Figure 2(b)). This was associated with a reduced expression of antibacterial response genes, indicating that a dysbiotic state in the mammary gland may promote cancer progression [34]. The idea that a specific breast microbiota can drive cancer pathogenesis is further supported by other studies [34, 35]. *Enterobacteriaceae* and *Staphylococcus* were recently found at higher abundance in breast cancer patients than in healthy controls [35]. *Escherichia coli* (*E. coli*) and *Staphylococcus epidermidis* (*S. epidermidis*) from normal adjacent tissue of breast cancer patients were found to induce DNA double-strand breaks (DSBs), which are the most detrimental type of DNA damage [35]. Recently, the analysis of 668 breast tumor tissues and 72 non-cancerous adjacent tissues by utilizing RNA sequencing data from the TCGA dataset demonstrated the prominent presence of Proteobacteria in tumor tissues [36]. Conversely, Actinobacteria prevail in non-cancerous adjacent tissues [36]. Overall, the existence of a specific microbiota is increasingly recognized to be associated with breast tissue, with some signatures found to discriminate among breast cancer subtypes [37] (Figure 2(c)). A mechanistic insight into the functional role of the microbiota in breast cancer pathogenesis is also emerging. Implementation of a semisupervised approach allowed revealing an association between microbial composition and tumor-specific gene expression profiles [36]. In particular, it was observed that *Listeria fleischmannii* is associated with genes involved in EMT, and *Haemophilus influenzae* is correlated with genes involved in the control of G2-M checkpoint, E2F signaling, and mitotic spindle assembly [36]. These functional associations gained further experimental support from other studies showing that various microbiota metabolites (such as the cadaverine produced by the gut microbiota) play a tumor suppressor role in breast cancer by reverting EMT and reducing cancer cell stemness, motility, and metastatic properties [38]. The molecular mechanisms appear to involve the interaction with trace amino acid receptors (TAARs), whose overexpression is associated with better survival of breast cancer patients [38].

The breast microbiota has a composition quite different to that of the gut and can exert its own independent effects on the breast tissue microenvironment. However, bacterial translocation from gut to breast does impact on breast cancer pathogenesis. Clinical studies revealed significant taxonomic differences in the gut microbiota between premenopausal breast cancer patients and healthy controls. These differences were associated with a specific microbiota composition and with an enrichment of inflammatory genes such as those modulating the synthesis of lipopolysaccharide (LPS) and butyrate [39]. A number of different mechanisms modulate gut dysbiosis-dependent breast cancer. First, gut bacteria influence estrogen metabolism [40, 41] (Figure 2(d)). Second, diet-derived processed metabolites that are produced in the gut are crucial for the regulation of the breast microbiome [42]. It has been recently demonstrated that the diet alone may modulate the mammary gland microbiota. For instance, consumption of Mediterranean diet results in a significant increase of *Lactobacillus* in the mammary gland, while this effect is not observed upon consumption of Western diet [43]. This appears of high importance in the process of breast carcinogenesis, as malignant breast tumors have lower *Lactobacillus* abundance than benign lesions, thus supporting a tumor suppressor role of *Lactobacillus* in breast cancer [43]. Third, the gut microbiota releases substances that influence EMT. Prominent examples include cadaverine, as discussed above, and the secondary bile acids product lithocholic acid (LCA). In breast cancer models, LCA inhibits cancer cell proliferation by regulating citric acid cycle (TCA) and oxidative phosphorylation (OXPHOS) through the G-protein-coupled bile acid receptor (Gpbar1) [44]. In addition, LCA affects lipid metabolism and apoptosis of breast cancer cells by activating protein kinase A (PKA) [45]. LCA levels were found reduced in the serum of breast cancer patients as compared with healthy individuals. The lower LCA production was associated with a reduction in both aerobic and anaerobic microbial populations [44]. The role of the microbiota on breast cancer progression appears extremely complex and deserves deeper investigations, as many bacteria (either pathogenic agents or harmless commensals) and conditions (e.g., nipple colonization by oral cavity bacteria during breastfeeding) can modulate breast eubiosis and eventually drive pathogenetic/malignant processes through induction of dysbiosis [46].

## 6. Esophagus

The interest on the esophageal microbiota remains limited as compared with other human tissues, primarily because the esophagus was considered as a channel connecting the oral cavity with the stomach. The first studies on esophageal microbiota demonstrated that the bacterial species found in the esophagus came from other districts, such as the oropharynx by swallowing or the stomach by reflux [47–49]. Gastroesophageal reflux disease (GERD) is a clinical condition where the esophagus is chronically exposed to acid, bile, and other stomach contents. GERD induces inflammation of the esophageal squamous epithelium and is generally thought to be the major cause of Barrett's esophagus (BE), a premalignant metaplasia of the squamous epithelium that could lead to esophageal adenocarcinoma (EAC) [48, 50–52]. Most autochthonic esophageal microbiota is non-culturable, so that the culture-based methods are prone to underestimate the complexity of these inhabitant commensals [47, 53]. Attempts to use cultures of aspirated esophageal washes showed *Streptococcus viridans* as the most frequent microorganism present in the normal esophagus and the oropharynx [47, 54, 55]. Culture-independent PCR analysis of the 16S rDNA from tissue biopsies allowed to discover an indigenous microbiota closely associated with the epithelial cell surface of the normal esophagus [56]. Six major phyla (Actinobacteria, Bacteroidetes, Firmicutes, Fusobacteria, Proteobacteria, and TM7) and 41 genera were identified; *Streptococcus*, *Prevotella*, and *Veillonella* were the most represented bacterial populations [56]. Distal esophageal mucosal biopsies from normal esophagus, GERD, and BE were then compared by 16S rRNA sequencing and cluster analysis [57]. Two types of microbiome were identified: the type I was mainly associated with normal esophagus and dominated by Gram-positive bacteria (*Streptococcus*); the type II was largely correlated with GERD and BE and composed by Gram-negative anaerobes/microaerophiles (*Veillonella, Prevotella, Haemophilus, Neisseria, Rothia, Granulicatella, Campylobacter, Porphyromonas, Fusobacterium,* and *Actinomyces*) [57]. Gall and coworkers recently found that *Streptococcus* and *Prevotella* dominate the upper gastrointestinal tract and the ratio of these two species can be associated with two known EAC risk factors in BE (waist-to-hip ratio and hiatal hernia length) [58]. Gram-negative bacteria are characterized by the presence of LPS in the outer membrane. LPS induces innate immune responses by upregulating proinflammatory cytokine genes through the Toll-like receptor (TLR) 4 and the downstream NF-*κ*B pathways. Activation of NF-*κ*B leads to production of IL-1*β* or TNF-*α* by inflammatory cells. This state of chronic inflammation is thought to play a crucial role in the progression from benign to malignant disease [47, 59–61]. Stepwise increased expression of IL-1*β*, IL-8, and NF-*κ*B was found to occur in the transition from normal esophageal epithelium to BE and EAC [62]. Gram-negative bacteria can reduce dietary nitrates to nitrites, which can be transformed into carcinogenic *N*-nitroso compounds in acidic environments [63]. Bile acids and nitrosamines induce an IL-1*β*- and IL-6-dependent inflammation, leading to BE and EAC [64]. The prognosis of esophageal squamous cell carcinoma (ESCC) has been positively associated to the presence of a periodontal major inhabitant, *F. nucleatum*, which could migrate from the oral cavity and colonize the esophagus; the mechanisms involve production and activation of various chemokines, such as CCL20 [65]. Recently, the microbial diversity in EAC tissues was found to be significantly reduced compared with control tissues, with an increased relative abundance of *Lactobacillus fermentum* [66]. Epithelial tissues in the oral cavity and the esophagus can be colonized by similar bacteria through the saliva. Therefore, it is expected that each bacterium would be present with the same abundance in the saliva and in esophageal cancer tissues. Unexpectedly, *Treponema denticola* and *Streptococcus anginosus*, which are minor bacteria in the saliva, were found to preferentially infect the normal mucosa of the esophagus as well as the esophageal cancer tissues [67]. The correlation between altered microbiota in the saliva and esophageal cancer was confirmed by other studies showing lower abundance of *Bulleidia, Lautropia, Catonella, Peptococcus, Moryella, Corynebacterium,* and *Cardiobacterium* and increased levels of colonization by *Prevotella, Streptococcus,* and *Porphyromonas* in ESCC *vs* normal tissues [68, 69]. This evidence appears of high interest, as *Streptococci* invade their target cells through a binding between the hyaluronic acid capsular polysaccharide on the bacterial surface and the hyaluronic acid-binding protein CD44 on the epithelial cells. The signaling pathway induced by *Streptococci* upon adhesion to CD44 involves cytoskeletal reorganization via Rac1 and ezrin, as well as loss of intercellular junctions by changed distribution of the junctional proteins ZO-1 and E-cadherin [70, 71]. Thus, loss of intercellular adhesions, as occurring upon EMT [72, 73], could mediate malignant progression in esophageal cancers.

## 7. Stomach

Before the discovery of *H. pylori*, the stomach was considered as a sterile organ, because of its thick mucus layer, the acidic gastric juice, and the peristaltic movement. *H. pylori* survives under the acidic conditions in the stomach, due to the action of a surface-exposed urease that transiently buffers the acidic environment by catalyzing the hydrolysis of the urea into carbon dioxide and ammonia. These two products serve as substrates for other microbes and can change the gastric microbiota. It is now well established that gastric cancer is associated with bacterial dysbiosis within the stomach and that chronic infection with *H. pylori* is a major risk factor for the development of gastric cancer. Although *H. pylori* infects >50% of the world's population and only ∼1% of the infected individuals develops gastric cancer, it is estimated that 75% of all gastric cancer cases are caused by infection with *H. pylori*, which is a class I carcinogen according to the World Health Organization. Colonization of the human stomach by *H. pylori* induces a complex inflammatory and immunological response, with production of TNF*α* and IL-1*β*, which is also a powerful inhibitor of gastric acid secretion [74]. This drives a cascade of events starting with chronic gastritis in virtually all infected individuals, which progresses from atrophy to intestinal metaplasia to dysplasia, and finally to gastric cancer [53, 75]. The molecular mechanisms of *H. pylori*-dependent gastric cancer have been elucidated in detail [76–82]. In the stomach, *H. pylori* localizes in close proximity to the apical cell-cell junctions and drives EMT through disruption of tight and adherens junctions [83–86]. The *H. pylori* cytotoxin-associated gene (*cag*) pathogenicity island encodes a type IV secretion system (T4SS) that translocates the effector protein CagA into the host cells after bacterial attachment to host apical or basolateral integrins [87]. Here, CagA associates with cell-cell junctions and recruits ZO-1 to its site of attachment, leading to altered assembly of these structures [83]. Once translocated into the host cells, CagA undergoes tyrosine phosphorylation by Src and Abl kinases within the repeated five amino acid motif EPIYA [88]. This phosphorylation is required to trigger EMT via upregulation of vimentin, Snail, Zeb1, CD44, and MMP-3 [80]. The phospho-CagA also binds the phosphatase SHP2 and the adaptor protein Grb2, inducing epithelial barrier dysfunctions and morphologic alterations such as cell elongation [88]. Nuclear accumulation of *β*-catenin is increased within gastric cancer precursor lesions such as gastric adenomas [89], suggesting a strong correlation of *β*-catenin with gastric cancer plasticity. Activation of *β*-catenin by CagA occurs through multiple mechanisms, including destabilization of E-cadherin/*β*-catenin complexes and activation of the Wnt signaling pathway [90], phosphorylation-independent modulation of the hepatocyte growth factor receptor (HGFR) [91], and degradation of GSK3*β* with activation of *β*-catenin and Snail [92]. *H. pylori*'s vacuolating cytotoxin A (VacA) and outer inflammatory protein A (OipA) can activate the epidermal growth factor receptor (EGFR) and the downstream PI3K-Akt signaling pathway, which results in GSK3*β* degradation and *β*-catenin stabilization [93, 94]. The infection of gastric epithelial cell lines by *cagA*-positive *H. pylori* strain induces morphological modifications characterized by a loss of polygonal shape, cell cluster disruption, elongated shape, stimulation of mesenchymal (Snail, vimentin, and Zeb1) and stem cell (CD44) markers, and inhibition of epithelial markers (cytokeratin 7 and osteopontin) [81]. Compared with CD44^low^ cells, *H. pylori*-infected CD44^high^ cells show mesenchymal phenotype and stem cell properties *in vitro*, high sphere-forming potential, enhanced migration and invasion capacities, and high tumorigenic capacity in xenografted mice [81]. The ability of CagA to subvert multiple host cellular pathways is also supported by other studies demonstrating that the activation of YAP signaling after *H. pylori* infection promotes gastric tumorigenesis [77].

## 8. Colon

Various models of bacteria-induced carcinogenesis have been postulated, suggesting how intestinal microbe-microbe and microbe-host interactions can contribute to development and progression of CRC [95]. A recent excellent article from Li et al. emphasized the importance of multispecies bacterial biofilms in modulating CRC progression [96]. Several pathogenic bacteria, such as *Bacteroides fragilis*, barely detectable in the normal intestinal microbiota may exhibit pro-oncogenic activity in CRC [95, 97]. It has been demonstrated that the microbial status modulates the development of colitis-associated CRC in the IL-10-deficient (*Il10*^*−/−*^) mouse model [98]. Administration of the colon-specific carcinogen azoxymethane (AOM) to germ-free *Il10*^*−/−*^ mice mono-associated with the mouse adherent-invasive commensal *E. coli* NC101 promoted invasive carcinoma in 80% of the hosts, while the human commensal *Enterococcus faecalis* OG1RF, which also caused aggressive colitis in *Il10*^*−/−*^ mice, was unable to induce malignant progression [99]. The molecular mechanism was demonstrated to involve the 54 kb polyketide synthase (*pks*) genotoxic island, which encodes a multienzymatic machinery that synthesizes colibactin, a peptide-polyketide hybrid [99, 100]. Colibactin induces DNA DSBs in the host cells and G2-M cell cycle arrest through the ATM → CHK2 → CDC25 C phosphorylation cascade [86].

The impact of the microbiota on the innate and adaptive immune system is well established [13, 101, 102]. In many epithelial tissues, the microbial communities are physically separated from the surrounding immune cells by an epithelial barrier [103]. In a mouse model of impaired intestinal barrier function, the exposure of immune cells to the microbiota was demonstrated to favor intestinal tumor growth through IL-23/IL-17 [103]. IL-23 is produced mainly by tumor-associated myeloid cells, which are able to penetrate the tumor but not the adjacent tissue, and can be activated by microbial products [103]. Accumulating data also support a role for the *B. fragilis* in inducing tumorigenesis in human and animal models of CRC. A recent study in germ-free *Apc*^*Min/+*^ mice colonized with *B. fragilis* showed that the *B. fragilis* toxin (BFT) induces the activation of a procarcinogenic, inflammatory cascade in intestinal epithelial cells, which requires the combined activation of the IL-17R and Stat3 signaling pathways [104]. IL-17R signaling in intestinal epithelial cells induces a proximal-to-distal gradient of chemokines, including CXCL1 [104], which leads to colon tumorigenesis through recruitment of CXCR2-expressing immature myeloid cells [104]. BFT was shown to bind a still unknown intestinal epithelial cell receptor, resulting in cleavage of E-cadherin and consequent shedding of its 80 kDa ectodomain. This leads to adherens junction disassembly, activation of the *β*-catenin/TCF signaling pathway, and enhanced cell proliferation [105, 106]. Chronic inflammation can sensitize hyperplastic tissues to transforming insults. Inhibition of the microRNA miR-34a, which protects the inflammatory stem cell niche, can lead to CRC after *Citrobacter rodentium* infection [107]. A key mechanism involves the activation of the Wnt/*β*-catenin, Notch, and TGF*β* pathways, together with epigenetic changes such as histone modification and chromatin remodeling. This triggers trans-differentiation of crypt cells into fibroblast-like mesenchymal cells [108].

Nonpathogenic intestinal commensals, such as *Lactobacilli* and *Bifidobacteria*, can be outcompeted by opportunistic/passenger bacteria better adapted to the tumor microenvironment of human CRC [95, 97]. The resulting altered microbiota stimulates further progression towards malignancy. An opportunistic bacterium with a key role in CRC progression is the Gram-negative anaerobic bacillus *F. nucleatum*. Its mechanistic contribution to CRC has been extensively documented, at variance with other human conditions such as pericarditis, brain abscesses, osteomyelitis, and other cancer types (oral, head and neck, and esophageal), where *F. nucleatum* appears to play a secondary role [86]. *F. nucleatum* is the most abundant bacterial species in the oral cavity, but it is a weak colonizer of the gut. The abundance of *F. nucleatum* in CRC tissues has been demonstrated using orthogonal approaches, such as 16S ribosomal RNA (rRNA) gene sequencing and fluorescent in situ hybridization [109]. At variance with *B. fragilis* and *E. coli*, that produce toxins able to change the immune response or to induce DNA damage, *F. nucleatum* is not known to produce toxins. Its major pro-oncogenic activities are due to the virulence factor Fusobacterium adhesin A (FadA), which is expressed on the bacterial surface, and whose corresponding gene *fadA* is strongly upregulated in colon tissue samples from patients with adenomas and adenocarcinomas as compared with healthy subjects [110]. FadA binds to and induces phosphorylation/internalization of E-cadherin and consequent disruption of cell-cell junctions [110]. Release of *β*-catenin from the plasma membrane and activation of Wnt signaling then occur, leading to enhanced cancer cell EMT and plasticity [86]. *F. nucleatum* can influence cancer progression through the creation of a proinflammatory niche. *Apc*^*Min/+*^ mice fed *F. nucleatum* developed more colorectal and small-intestinal tumors than their sham-fed counterparts [111]. It was found that *F. nucleatum* induces intratumoral myeloid cells, including macrophages, dendritic cells, and myeloid-derived suppressor cells [111]. *F. nucleatum* also activates the NF-*κ*B pathway and induces expression of the genes encoding several pro-inflammatory cytokines, such as TNF*α*, IL-6, IL-8, and IL-1*β* [111]. Analysis of surgically treated stage III/IV CRC patients showed that the levels of *F. nucleatum* are significantly higher in tumor tissues than in adjacent normal tissues, and correlate with tumor invasion as well as with lymph node and distant metastasis [112]. This supports the role of *F. nucleatum* in promoting CRC progression, possibly through the formation of an oncogenic biofilm [96]. Recent findings showed that *F. nucleatum* induces loss of miR-4802 and miR-18a, with consequent increased expression of the autophagy signaling elements ULK1 and ATG7 [113]. This results in enhanced chemoresistance through activation of the innate immune signaling pathways dependent on TLR4 and MYD88 [113]. A positive correlation between *F. nucleatum* levels in CRC tissues and the expression levels of Nanog was observed, supporting a role of *F. nucleatum* in modulation of cancer plasticity and EMT [112].

## 9. Female Reproductive Tract

The female reproductive tract (cervical canal, uterus, fallopian tubes, peritoneal fluid, and vagina) harbors complex and diverse bacterial communities that are associated with different physiological and pathological conditions [114, 115]. Members of the genus Lactobacillus are commonly identified as the hallmark of normal vagina with a major role in protecting the vaginal environment from colonization by other pathogenic organisms. This prevents bacterial vaginosis, yeast infections, and sexually transmitted diseases [116, 117]. Evidence of an altered microbiota associated with cancer came from studies in endometrial, vaginal, and ovarian cancer tissues. These studies demonstrated that *Atopobium vaginae* and *Porphyromonas* sp. in the gynecologic tract, in combination with a high vaginal pH (>4.5), are associated with the presence of endometrial cancer [118]. Thus, vaginal infections may cause chronic upper genital tract infection and inflammation and trigger carcinogenesis. A reduced relative abundance of *Lactobacillus* spp. causes increased production of several inflammatory cytokines, such as IL-36*γ*, MIP-1*β*, RANTES, IP-10, IL-2, IL-4, Flt-3L, and sCD40L [119]. Alterations of the vaginal microbial community have been also proved to impact pregnancy, bacterial vaginosis, and carcinogenesis [120]. A correlation between the human papillomavirus- (HPV-) induced cervical cancer and altered homeostasis of microbial vaginal communities was observed [121–123]. In cervical cancer, disease severity and high-risk HPV persistence are associated with increased diversity of the vaginal microbiota, suggesting a possible causal relationship with cancer onset and progression [121, 123].

Ovarian cancer accounts for about 3% of cancers among women and is the most lethal gynecological malignancy. Evidence from two different studies indicated that the ovarian cancer microbiota is distinctly different from that of the normal ovarian tissue. Persistent infection of ovarian tissues with *Proteobacteria* and *Firmicutes* may strongly reduce microbial diversity and lead to ovarian tumorigenesis by suppressing host inflammatory immune responses in the tumor microenvironment [124, 125].

## 10. Skin

The skin is a complex ecosystem inhabited by bacteria, archaea, fungi, and viruses. The microbiota is fundamental to skin physiology and immunity, and interactions between skin microbes and the host are quite complex and context-dependent, ranging from mutualistic to pathogenic relationships [126]. Types and numbers of bacteria (and other microorganisms) that colonize the human skin are determined by several distinct host characteristics (i.e., age and ethnicity) and lifestyles (i.e., hygienic routine, topical medications, and/or cosmetics). A key role is also played by genetic factors or systemic/local diseases (such as immunodeficiency syndromes and dermatitis) and by environmental determinants (i.e., humidity and geographic location). Across skin regions, glands and hair follicles provide distinct niches for growth of specific microbial communities. For example, *Cutibacterium* and *Staphylococcus* are prevalent colonizers of sebaceous areas, while *Corynebacterium*, *Staphylococcus*, and *beta-Proteobacteria* are found in moist areas. Skin cancers are generally classified into two main groups, melanoma and non-melanoma skin cancers (NMSC), having different etiology and clinical behavior. NMSC arises from epidermal keratinocytes and can be divided into basal cell carcinoma (BCC) and squamous cell carcinoma (SCC). Melanoma develops from epidermal melanocytes. Overall, NMSC shows higher incidence than melanoma, but a better response to treatment and better long-term prognosis. While the role of viruses and environmental carcinogens (such as UV) in skin cancer progression has been deeply elucidated, the contribution of the bacterial microbiota remains controversial [127], though a reduced rate of skin cancer in germ-free rats was observed [128]. Chronic inflammatory skin diseases, such as psoriasis, have been associated with development of skin cancers [129]. Injured skin of psoriasis patients has been found to host an altered microbiota, with increased abundance of *Firmicutes* and *Actinobacteria* [130]. Certain strains of *S. aureus* have been implicated in the pathogenesis of atopic dermatitis [131], which was recently correlated with increased risk of developing BCC in male patients [132]. Interestingly, an altered gut microbiota, in addition to having cancer-promoting effects within the gastrointestinal tract, has been recently reported to be associated with cancers of other organs including skin [127]. Also, gut dysbiosis has been correlated with several skin diseases, including acne vulgaris by increased immune response to some species of gut bacteria. Of note, oral probiotics such as *Lactobacillus paracasei* have been found to be beneficial for the skin due to their immunomodulatory effects [133, 134]. Chronic inflammation may have a causal role in the microbiota-modulated skin carcinogenesis. *S. epidermidis* was found to protect against UVB-induced skin papillomas in preclinical models [135]. *S. epidermidis* produces 6-*N*-hydroxyaminopurine (6-HAP), which showed a bactericidal activity against pathogens such as the group A *Streptococcus* (GAS) by inhibiting DNA polymerase [135]. In culture, 6-HAP selectively inhibits the proliferation of tumor cell lines but does not inhibit primary keratinocyte growth. Thus, some members of the skin microbiota may suppress tumor growth, and dysbiosis is potentially detrimental because induces loss of a protective function rather than gain of a detrimental microbial community [135].

## 11. Are There Common Mechanisms of Microbiota-Induced Tumor Plasticity?

### 11.1. Microbiota-Triggered EMT

The acquisition of motile/mesenchymal properties by epithelial cells is the phenotypic effect of a profound molecular and cellular reprogramming [136–139]. The cancer microbiota impacts EMT and its reversal process MET by acting on several signaling pathways. As EMT/MET activation has clinical implications for cancer progression and prognosis, elucidating these mechanisms will be critical to the development of novel targeted therapies. Due to the complexity of the networks potentially affected by specific bacterial inhabitants, a challenge will be to develop novel technological approaches for molecular detection and bioinformatic analysis of signaling pathways potentially affected by dysbiosis. For instance, mass spectrometry-based phosphoproteomics integrated with transcriptomics are allowing to build up a comprehensive map of proteins, including several EMT factors, modulated after *Chlamydia trachomatis* infection [140].

**Figure 3:**
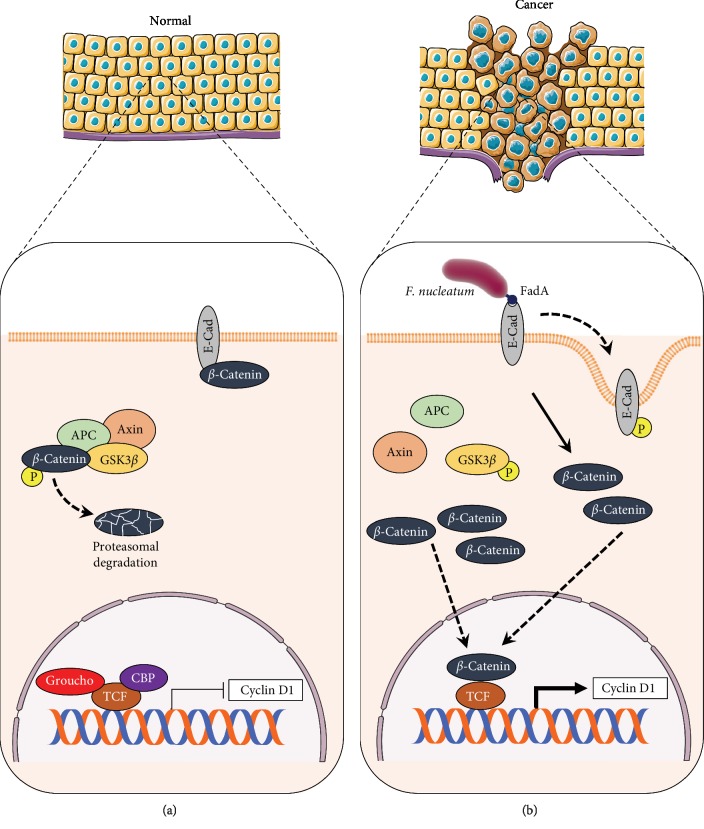
Microbiota-triggered EMT via E-cadherin/*β*-catenin. (a) In normal epithelial tissues, *β*-catenin activity is kept at low levels by degradation of the *β*-catenin cytoplasmic pool upon binding to the APC/Axin/GSK3*β* complex, or by membrane retention of *β*-catenin by interaction with E-cadherin. (b) Opportunistic infections by various pathogens, such as *F. nucleatum* in the human colon, can promote malignant progression. The virulence factor FadA is expressed on the surface of *F. nucleatum*. It binds to and induces phosphorylation/internalization of E-cadherin with consequent disruption of cell-cell junctions. Release of *β*-catenin from the plasma membrane and further activation of the Wnt signaling pathway (phosphorylation/degradation of GSK3*β* and disassembly of the APC/Axin/GSK3*β* complex) then occur, leading to enhanced cancer cell EMT and invasion. Top panels have been modified from Servier Medical Art, licensed under a Creative Common Attribution 3.0 Generic License; http://smart.servier.com/.

A growing body of evidence suggests that the cancer microbiota can promote tumorigenesis and plasticity through metabolic reprogramming [38, 44, 141]. A defining feature of EMT is to enhance resistance to anoikis [142]. This can be obtained in malignant cells through suppression of mitochondrial oxidative phosphorylation; specific EMT factors, such as Snail, can play a major role by regulating glucose metabolism via cytochrome C oxidases [143]. However, most EMT-promoting pathogens act by directly inhibiting intercellular adhesions between epithelial cells upon attachment to the mucosal layers. As described in the previous sections, infection of the oral mucosa by *P*. * gingivalis* leads to overexpression of EMT factors such as Zeb1 [20]. Bacterial adhesins bind to cell-cell proteins including E-cadherin and regulate cell polarity and downstream signaling pathways. This was reported for *F. nucleatum*, which promotes CRC by modulating E-cadherin/*β*-catenin signaling through the adhesin FadA [110] (Figure 3). The *H. pylori* protein CagA can subvert multiple signaling cascades into host epithelial cells [85]. Although CagA and other virulence factors including VacA are the most studied protumorigenic factors [144], several noncanonical, CagA-independent mechanisms of gastric carcinogenesis have also been reported [76, 145]. Dependent and independent mechanisms all converge on the activation on EMT through displacement and downregulation of cell-cell junction proteins.

### 11.2. Microbiota-Dependent, Tumor-Promoting Inflammation

As described in the previous sections, the role of the microbiota in cancer progression is largely connected to the modulation of host inflammatory responses in many body sites. In the lung parenchyma, the microbiota promotes production of IL-1*β*, IL-23, and IL-17 by myeloid and *γδ* T cells [33]. Gram-negative pathogens such as *H. pylori* play a major role in inducing inflammation-dependent carcinogenesis, as they stimulate host production of IL-1*β* and TNF-*α* [47, 59–61, 74]. In the intestinal mucosa, *B. fragilis* induces an inflammatory cascade through IL-17R and Stat3 [104]. This stimulates a CXCL1 gradient [104], which recruits immature myeloid cells and promote CRC [104]. Moreover, *F. nucleatum* stimulates the production of TNF*α*, IL-6, IL-8, and IL-1*β* [111], and infection of the urogenital tract with *Lactobacillus* spp. determines increase of various inflammatory cytokines, including IL-2 and IL-4 [119]. Activation of the EMT program in cancer cells has been correlated with infiltrating tumor-associated macrophages (TAMs), which are often re-educated by the tumor microenvironment to support extracellular matrix remodeling, angiogenesis, immunosuppression, and acquisition of invasive properties instead of eliminating cancer cells [146]. TAMs produce soluble growth factors (i.e., HGF, EGF, TGF*β*, PDGF, etc.) and inflammatory cytokines (IL-1*β*, IL-6, and TNF*α*) that can induce EMT in cancer cells. Also, myeloid cells have been shown to induce EMT-like properties in cancer cells via TGF*β*, EGF, and HGF [146]. Chronic inflammation-associated immunosuppression by regulatory dendritic cells (DCregs) and regulatory T cells (Tregs) is strongly correlated with induction of EMT [147]. It has been recently reported that the use of broad-spectrum antibiotics shortly before or after the initiation of PD-1/PD-L1 blockade can be associated with poor clinical outcome in cancer patients [148], thus indicating that the microbiota plays a central role in the cancer immunosurveillance [149]. The molecular mechanisms may involve a cross-regulatory loop between EMT and immunosuppression through the miR-200/Zeb1 axis, that directly controls the expression of PD-L1 on tumor cells, and the consequent effector T-cell exhaustion [147].

## 12. Concluding Remarks and Perspectives

Dysbiotic states where the amount of pathogenic inhabitants overrides or even replaces the non-pathogenic commensals can have detrimental effects on the physiological processes and lead to various types of diseases, including cancer (Table 1). Two common mechanisms appear largely modulated by the pathogenic microbial communities to induce tumorigenesis: epithelial barrier alteration with induction of EMT, and tumor-promoting inflammation. Elucidating the molecular mechanisms that underlie the enhanced cancer cell plasticity induced by an altered microbiota will allow the development of new strategies for targeted therapy. Restoring the microbial populations through fecal microbiota transplantation [150] or by treatment with microbial modulators such as the high-affinity polyreactive IgA [151] has demonstrated remarkable efficacy in some conditions, such as recurrent/refractory *Clostridium difficile* infection [150] or lymphoproliferative disease/ulcerative colitis [151]. This approach holds a therapeutic potential for treatment of various cancers and associated diseases, through reconstitution of the physiological microbiota, improvement of bile acid metabolism, and strengthening of immunotherapeutic approaches.

## Figures and Tables

**Table 1:** Main identified/studied bacterial phyla and their relationship with carcinogenesis.

Bacterial Phylum	Body site	Detrimental/beneficial for the host	Reference
*Actinobacteria*	Colon	Beneficial (though outcompeted by opportunistic bacteria during cancer progression)	[95, 97]
	Esophagus	Beneficial	[68, 69]

*Bacteroidetes*	Colon	Detrimental, linked to development of CRC	[95, 97]
	Esophagus	Detrimental, linked to development of esophageal cancer	[57, 68, 69]
	Female reproductive tract	Detrimental, linked to endometrial cancer	[118]
	Oral cavity	Detrimental, related to development of OSCC	[18–20]

*Firmicutes*	Breast	*Staphylococcus*: detrimental, linked to development of breast cancer *Lactobacillus*: beneficial	[37, 43]
	Colon	Beneficial (though outcompeted by opportunistic bacteria during cancer progression)	[95, 97]
	Esophagus	Detrimental, linked to development of esophageal cancer *Peptococcus*: beneficial	[57, 68, 69]
	Female reproductive tract	Detrimental, linked to ovarian cancer *Lactobacillus*: beneficial	[119, 124, 125]
	Nasal cavity	Detrimental, related to development of nasal polyposis	[28, 29]
	Skin	Detrimental, linked to atopic dermatitis and BCC (*S. aureus*) or beneficial (*S. epidermidis*)	[131, 135]

*Fusobacteria*	Colon	Detrimental, linked to development of CRC	[86, 110–112]
	Esophagus	Detrimental, linked to development of esophageal cancer	[57]
	Oral cavity	Detrimental, related to development of OSCC	[16, 17]

*Proteobacteria*	Breast	Detrimental, linked to development of breast cancer	[37]
	Esophagus	Detrimental, linked to development of esophageal cancer	[53, 57, 75]
	Female reproductive tract	Detrimental, linked to ovarian cancer	[124, 125]

## Abbreviations

*H. pylori*: *Helicobacter pylori*
P. gingivalis: *Porphyromonas gingivalis*
F. nucleatum: *Fusobacterium nucleatum*
*S. aureus*: *Staphylococcus aureus*
*E. coli*: *Escherichia coli*
*S. epidermis*: *Staphylococcus epidermidis*
HMP: Human microbiome project
EMT: Epithelial-mesenchymal transition
OSCC: Oral squamous cell carcinoma
MMP: Matrix metalloproteinase
SAE: Staphylococcal enterotoxins
ZO-1: Zonula occludens-1
LUAD: Lung adenocarcinoma
IL: Interleukin
TAAR: Trace amino acid receptor
LPS: Lipopolysaccharide
LCA: Lithocholic acid
TCA: Citric acid cycle
OXPHOS: Oxidative phosphorylation
Gpbar1: G-protein-coupled bile acid receptor
PKA: Protein kinase A
GERD: Gastroesophageal reflux disease
BE: Barrett's esophagus
EAC: Esophageal adenocarcinoma
ESCC: Esophageal squamous cell carcinoma
TLR: Toll-like receptor
NMSC: Nonmelanoma skin cancers
BCC: Basal cell carcinoma
SCC: Squamous cell carcinoma
6-HAP: 6-*N*-hydroxyaminopurine
GAS: Group A *Streptococcus*
cag: Cytotoxin-associated gene
T4SS: Type IV secretion system
VacA: Vacuolating cytotoxin A
OipA: Outer inflammatory protein A
EGFR: Epidermal growth factor receptor
HGFR: Hepatocyte growth factor receptor
CRC: Colorectal cancer
AOM: Azoxymethane
pks: Polyketide synthase
DSBs: Double-strand breaks
BFT: *B. fragilis* toxin
FadA: Fusobacterium adhesin A
TAMs: Tumor-associated macrophages
DCregs: Regulatory dendritic cells
Tregs: Regulatory T cells.
